# *In Vitro* Bioactivity Study of RGD-Coated Titanium Alloy Prothesis for Revision Total Hip Arthroplasty

**DOI:** 10.1155/2016/8627978

**Published:** 2016-07-14

**Authors:** Zhentao Man, Dan Sha, Shui Sun, Tao Li, Bin Li, Guang Yang, Laibo Zhang, Changshun Wu, Peng Jiang, Xiaojuan Han, Wei Li

**Affiliations:** ^1^Department of Joint Surgery, Shandong Provincial Hospital Affiliated to Shandong University, Jinan, Shandong Province 250021, China; ^2^Department of Medical Oncology, Shandong Provincial Hospital Affiliated to Shandong University, Jinan, Shandong Province 250021, China; ^3^Department of Orthopaedics, Jinan Central Hospital Affiliated to Shandong University, Jinan, Shandong Province 250013, China; ^4^Department of Neurology, Shandong Provincial Hospital Affiliated to Shandong University, Jinan, Shandong Province 250021, China

## Abstract

Total hip arthroplasty (THA) is a common procedure for the treatment of end-stage hip joint disease, and the demand for revision THA will double by 2026. Ti6Al4V (Titanium, 6% Aluminum, and 4% Vanadium) is a kind of alloy commonly used to make hip prothesis. To promote the osseointegration between the prothesis and host bone is very important for the revision THA. The peptide Arg-Gly-Asp (RGD) could increase cell attachment and has been used in the vascular tissue engineering. In this study, we combined the RGD with Ti6Al4V alloy using the covalent cross-linking method to fabricate the functional Ti6Al4V alloy (FTA). The distribution of RGD oligopeptide on the FTA was even and homogeneous. The FTA scaffolds could promote mouse osteoblasts adhesion and spreading. Furthermore, the result of RT-qPCR indicated that the FTA scaffolds were more beneficial to osteogenesis, which may be due to the improvement of osteoblast adhesion by the RGD oligopeptide coated on FTA. Overall, the FTA scaffolds developed herein pave the road for designing and building more efficient prothesis for osseointegration between the host bone and prothesis in revision THA.

## 1. Introduction

Total hip arthroplasty (THA) is a common and effective procedure for the treatment of end-stage arthritic hip joint, more than 1 million of which were undertaken around the world [1]. And the incidence of primary THA is estimated to grow by 174% by 2030 because of the ageing population, while the demand for revision THA will double by 2026 [2].

The use of cementless hip prothesis is the current trend in total hip arthroplasty, because the cemented hip prothesis showed unacceptable loosening rates [3]. Secure bone integration between the host bone and prothesis is critical for the long-term survival of cementless hip prothesis [4]. Ti6Al4V (Titanium, 6% Aluminum, and 4% Vanadium) is a kind of alloy commonly used to make hip implant structures [5].

For the revision THA, severe femoral bone loss often took place due to osteolysis, aseptic femoral loosening, stress shielding, and so forth [6]. Sheth et al. have reported that bone loss and the poor integrity of the remaining bone stock are common complications in revision THA [7]. Hence, it is very important to furtherly promote the osseointegration between the host bone and prothesis [4, 8], especially for the protheses used for the revision THA accompanied by bone loss. Currently, lots of biological techniques have been developed to further improve the implant surface to increase the osseointegration between the host bone and prothesis in the revision THA [9], such as cell seeding and local release of bone morphogenic proteins [5]. Previous studies have reported that embryonic stem cells, fetal osteoblasts, and mesenchymal stem cells could be used to seed onto the metal prothesis to improve osseointegration [10–12].

The peptide Arg-Gly-Asp (RGD), which is derived from fibronectin in the extracellular matrix (ECM), has been confirmed to increase cell attachment [13, 14]. And also, the RGD peptides have stable bioactivity to resist harsh treatments [13], which have been used in the vascular tissue engineering to improve the functions of seed cells [15]. However, in bone tissue engineering, Anselme has reported that the adhesion and spreading of cells on the protheses are the first phase of cell/protheses interactions and the quality of this step will affect the subsequent capacity for cell proliferation and differentiation [16].

Therefore, in this study, we attempted to combine the RGD with Ti6Al4V alloy using the covalent cross-linking method to fabricate the functional Ti6Al4V alloy (FTA) and meanwhile determine the biological effect of FTA on osteoblast cells.

## 2. Materials and Methods

### 2.1. The Synthesis of Peptide and Preparation of Ti6Al4V Alloy

The peptide sequence of RGD was synthesized by the Scilight-Peptide Inc. based on the previous study [14]. And the RGD peptide was labeled with fluorescein-5-isothiocyanate (FITC) to facilitate the following detection.

Many scaffolds (5 mm × 5 mm) made of Ti6Al4V alloy were obtained from the surface of hip joint prothesis with a diamond band saw (Exakt, Apparatebau, Germany).

### 2.2. The Preparation of RGD-Coated Ti6Al4V Alloy

The functional Ti6Al4V alloy (FTA) scaffolds were prepared by covalent conjunction of RGD with the Ti6Al4V alloy specimens. The procedure of covalent conjunction was performed according to the previous report with some modifications [17]. Briefly, the Ti6Al4V alloy specimens (5 mm × 5 mm) obtained from hip joint protheses were immersed in 1 mL of 10% (w/v) 1,6-hexanediamine for 60 min at 37°C. After gently washing with distilled water, the specimens were soaked in 2 mg/mL sulfosuccinimidyl-4-(N-maleimidomethyl)cyclohexane-1-carboxylate (sulfo-SMCC, Thermo Fisher Scientific Inc., Rockford, IL, USA) solution for 1 h at room temperature. Subsequently, the specimens were immersed in 0.1 mg/mL RGD peptide solution for 12 h at 37°C. Ultimately, the RGD-coated Ti6Al4V alloy was freeze dried and UV sterilized (30 min) before use.

### 2.3. Detecting the Effect of Conjunction between RGD and Ti6Al4V Alloy

In order to determine the effect of conjunction between peptide and Ti6Al4V alloy, the RGD-coated Ti6Al4V alloy was observed under confocal microscope. The excitation and emission wavelengths were 488 and 525 nm, respectively [18].

### 2.4. Primary Osteoblast Cells Isolation and Culture

The animal experiments were approved by the local ethics commission complying with the “Guide for the Care and Use of Laboratory Animals” (NIH Publication number 85-23, revised 1996).

The culturing of osteoblast cells was performed according to the previous reports [19, 20]. Briefly, the femora and tibia were aseptically obtained from skeletally mature mouse; and then the long bones were minced into small pieces (1 mm^3^). Subsequently, the minced bone tissues were repeatedly digested by 300 active U/mL collagenase solution (Sigma-Aldrich, St. Louis, MO, USA) and incubated with EDTA solution. For each digestion, the cell suspensions were aspirated and centrifuged to harvest the osteoblasts, which were cultivated with Dulbecco's minimal essential medium (DMEM) supplemented with 10% fetal bovine serum (FBS, HyClone, Logan, UT, USA), 2 mM L-glutamine, and 100 U/mL penicillin/streptomycin in culture dishes. And osteoblasts at passage 2 were used in the following experiments.

### 2.5. Osteoblasts Seeding on Different Scaffolds

When passage 2 osteoblasts had 80% confluence in culture dishes, the osteoblasts were digested with 0.25% trypsin-EDTA and washed twice with DMEM. Subsequently, the osteoblasts (at a density of 3.0 × 10^5^ cells/cm^2^) were seeded on two different groups of scaffolds: one group used the functional Ti6Al4V alloy (FTA), which was coated with RGD by covalent cross-linking, and the other group used common Ti6Al4V alloy (CTA), which served as the control group. Subsequently, the osteoblast-loaded scaffolds were cultured with the DMEM, and the specimens were harvested at specific time points for different analyses.

### 2.6. Determining the RGD Function by Counting the Cell Number

To determine the effect of RGD, the cell distribution and number on FTA or CTA scaffolds were separately studied after 7 h of culturing [17, 21, 22]. To observe the distribution of osteoblasts on different scaffolds, the osteoblast-loaded scaffolds were washed twice with PBS to remove unattached cells. Subsequently, the scaffolds were fixed with 4% paraformaldehyde for 15 min and stained with Hoechst 33258 for 5 min. Finally, the distribution of osteoblasts in different groups was observed under a confocal microscope with the wavelengths of 360 and 460 nm for excitation and emission, respectively.

To further analyze the cell number, the osteoblast-loaded scaffolds were washed twice with PBS to remove unattached cells after 7-hour incubation. Subsequently, the cells attached on the scaffolds were retrieved by the incubation of 0.25% trypsin-EDTA for 20 min. Finally, cell number was determined using the Automated Cell Counter (Scepter) [17].

### 2.7. Determining the RGD Function by Observing the Morphology of Osteoblasts

To further determine the effect of RGD, the spreading morphology of osteoblasts was observed [17, 23]. Briefly, after culturing for 24 h, the osteoblast-loaded scaffolds in both FTA group and CTA group were fixed with 4% paraformaldehyde for 15 min. Subsequently, the samples were incubated with rhodamine phalloidin (Cytoskeleton Inc., Denver, CO, USA) for 1 h at 37°C to label the cytoskeleton. Finally, the spreading morphology of osteoblasts on different scaffolds was observed under a confocal microscope with the wavelengths of 488 and 517 nm for excitation and emission, respectively.

### 2.8. Determining the Capacity of Osteogenesis on Different Scaffolds

The capacity of osteogenesis by the osteoblasts in the FTA group or CTA group was determined with real-time qPCR. The gene expressions of alkaline phosphatase (ALP) and collagen I (COL1) were chosen as the markers [24]. After 7 days of culture, the osteoblast-loaded specimens were harvested for the determination of real-time qPCR, and the operation was performed on an Applied Biosystems 7300 with the following oligonucleotide primers: ALP, forward primer: 5′-CCACGTCTTCACATTTGGTG-3′; reverse primer: 5′-AGACTGCGCCTGGTAGTTGT-3′; COL1, forward primer: 5′-ACAGCCGCTTCACCTACAGC-3′; reverse primer: 5′-GTTTTGTATTCAATCACTGTCTTGCC-3′; and GAPDH, forward primer: 5′-GAGTCAACGGATTTGGTCGT-3′; reverse primer: 5′-TGGGATTTCCATTGATGAAC-3′. The relative change of gene expression was calculated using 2^−ΔΔCt^ method with GAPDH as the reference [25, 26].

### 2.9. Statistical Analysis

All experiments were performed in triplicate. The data were analyzed using Student's *t*-test by the SPSS 19 software. The results were expressed as mean ± standard deviation. *P* values less than 0.05 were considered statistically significant.

## 3. Results 

### 3.1. Characterization of RGD-Modified Ti6Al4V Alloy

**Figure 1:**
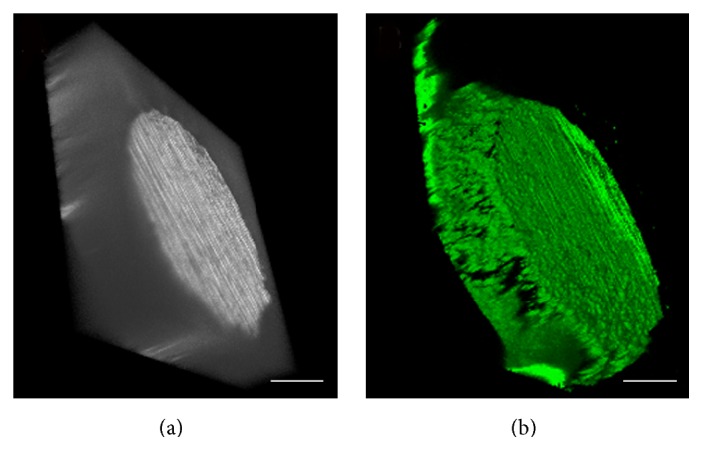
Confocal microscopy images of functional Ti6Al4V alloy. The homogenous green fluorescence illustrates that the RGD-FITC could be successfully and homogeneously conjugated onto the surface of Ti6Al4V alloy (bar = 400 *μ*m).

As shown in Figure 1, the green fluorescence of the FTA was even, which showed that the FITC labelled RGD could be successfully and homogeneously conjugated onto the Ti6Al4V alloy. This data indicated that our method of covalent cross-linking was useful.

### 3.2. Function of the RGD Determined by Osteoblast Counting

**Figure 2:**
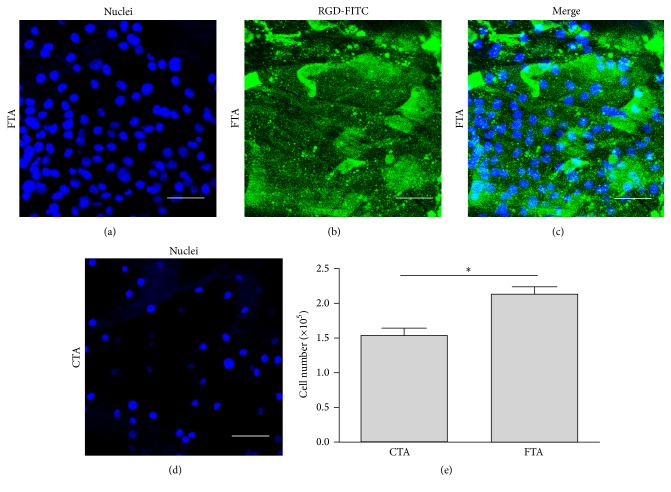
(a–d) The distribution of osteoblasts on different scaffolds. The cell density on the FTA scaffold (a–c) was superior to the CTA scaffold (d). Blue, nuclei staining with Hoechst 33258; green, RGD-FITC coated on the surface of Ti6Al4V alloy (bar = 50 *μ*m). (e) Quantitative analysis of cell number in different groups (*n* = 3; ^*∗*^ *P* < 0.05).

To determine the bioactivity of RGD, the cell number adhered on FTA or CTA scaffolds was analyzed. As shown in Figure 2, more osteoblasts were adhered onto the FTA after 7 h of incubation. The result of Student's *t*-test revealed that the cell number in the FTA groups was significantly higher than that in the CTA group (*P* < 0.05). From these data, we can conclude that the RGD coated on the surface of Ti6Al4V alloy could effectively promote the osteoblast adhesion.

### 3.3. RGD Coated on the Ti6Al4V Alloy Promoting Osteoblast Spreading

**Figure 3:**
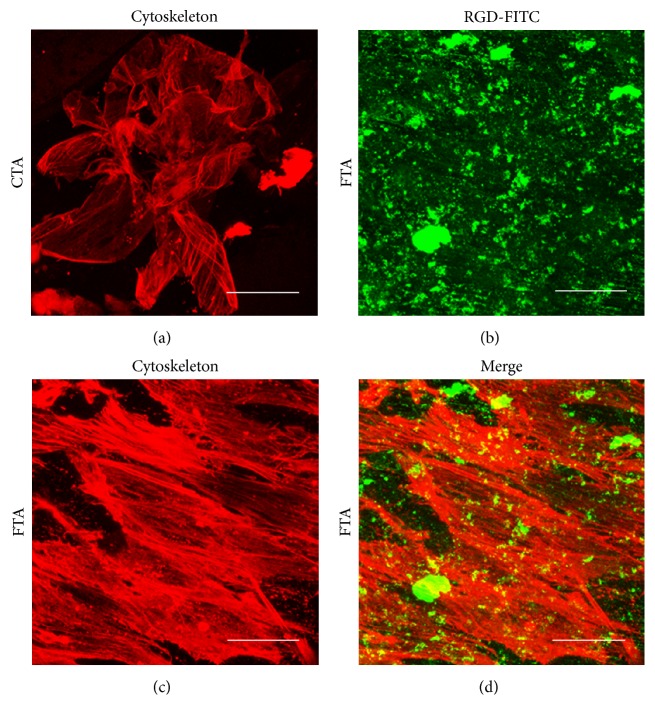
Osteoblasts spreading morphology in different groups after 24-hour incubation. Osteoblasts in the CTA group spread poorly (a), but osteoblasts in the FTA group exhibited normal spreading morphology (b–d). Red, cytoskeletal staining with rhodamine phalloidin; green, RGD-FITC coated on the surface of Ti6Al4V alloy (bar = 50 *μ*m).

To further observe the performance of seed cells on different scaffolds, the cytoskeletal staining of osteoblasts was performed with rhodamine phalloidin. As shown in Figure 3, the osteoblasts in the FTA group exhibited better spreading morphology than that in the CTA group, which indicated that the RGD could promote the osteoblast-scaffold interaction.

### 3.4. Capacity of Osteogenesis on Different Scaffolds

**Figure 4:**
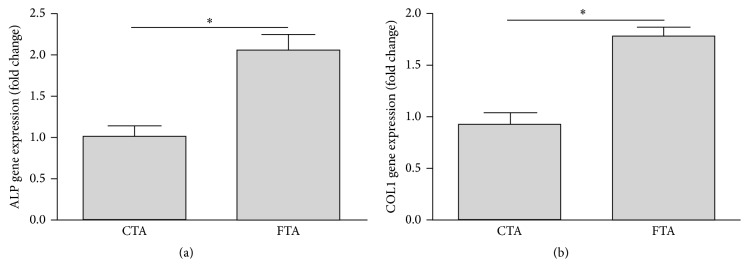
Real-time qPCR analysis of ALP and COL1 gene expression in different groups after 7-day incubation (*n* = 3; ^*∗*^ *P* < 0.05).

As shown in Figure 4, more ALP and COL1 genes were expressed in the FTA group than those in the CTA group after 7-day incubation. These data illustrated that the FTA scaffolds were more favorable for osteogenesis, which may be due to the improvement of osteoblast adhesion by the RGD peptide coated on the Ti6Al4V alloy.

## 4. Discussion

The revision THA has consumed 19% of the Medicare hip replacement expenditures between 1997 and 2003 in the USA, but the demand for revision THA will double by 2016 [2]. For revision THA, bone loss and the poor integrity between the remaining bone stock and hip prothesis are common complications [7, 27]. Currently, the Ti6Al4V alloy is a kind of alloy commonly used to make hip implant structures [5]. Previous studies have confirmed that the early strong adhesion between host bone and hip prothesis is very important [13].

However, in the revision THA, there must be some unmatched areas between the hip prothesis and its surrounding host bone due to the bone loss. Hence, there is a pressing need to find functional hip prosthesis materials to promote osteogenesis and further improve the osseointegration between the host bone and prothesis. Lots of biological techniques have been developed to further improve the implant surface to increase the osseointegration between the host bone and prothesis [5], such as the seeding of embryonic stem cells, fetal osteoblasts, or mesenchymal stem cells onto the metal implants [10–12].

The process of osseointegration between the prothesis and host bone belongs to the direct healing, which forms a direct structural and functional bone connection [28, 29]. The osseointegration was activated by the lesion of bone matrix via the release of noncollagenous proteins and growth factors [30]. Significantly, the osteoblasts begin to proliferate and synthesize bone matrix in bone defects. Therefore, attracting more osteoblasts onto the surface of prothesis might promote the osseointegration within the bone/prosthesis interface, especially for the prothesis used in revision THA.

The RGD is a kind of peptide derived from the ECM, which has been used in the vascular tissue engineering to improve the functions of seed cells [15, 31, 32]. In this study, we attempted to conjugate the RGD oligopeptide onto the surface of Ti6Al4V alloy using the method of covalent cross-linking to further improve the osseointegration between the host bone and prothesis.

As shown in Figure 1, we have successfully conjugated the RGD oligopeptide onto the surface of Ti6Al4V alloy, and the RGD distribution was even and homogeneous. From these data, we could conclude that the method of covalent cross-linking was mature and reliable.

The conjunction of RGD onto the Ti6Al4V alloy could enhance the adhesion of osteoblasts (Figure 2). The RGD oligopeptide is composed of arginine, glycine, and aspartic acid [33]. And the main effect of RGD is based on the binding to many members of the integrin family, which are the most numerous and versatile cell adhesion receptors [34]. The integrin family comprises distinct 24 *αβ* heterodimers, and the types of a3b1, a5b1, a8b1, aIIbb3, avb1, avb3, avb5, avb6, and avb8 could bind to RGD in a dependent manner [33, 35]. In this study, the RGD on the FTA may bind to the integrins in the osteoblasts and cause a cascade of biological events, which promote the adhesion of osteoblasts.

And, meanwhile, the osteoblasts in the FTA group have the superior spreading morphology. Previous studies have confirmed that the integrin could promote cell attachment, cell spreading, organization of cytoskeleton, and formation of focal adhesions [33]. In this study, the RGD on the surface of FTA may activate the integrin receptor of the osteoblasts and further improve the spreading morphology of osteoblasts in the FTA group (Figure 3). However, the specific biological mechanism for the RGD on the surface of FTA scaffolds would be comprehensively analyzed in our future study.

In addition to the state of better initial adhesion and spreading, the ALP and COL1 gene expressions in the FTA group were significantly higher than those in the CTA group (Figure 4). The seed cells used in this study were osteoblasts, which could maintain the expression of osteogenic markers. Previous studies have reported that the better initial adhesion of seed cells could promote the subsequent bioactivity of seed cells [17]. In this study, the better initial adhesion and spreading of osteoblasts in the FTA group are favorable for the ALP and COL1 gene expressions.

In brief, the fabrication of FTA by covalent cross-linking of RGD oligopeptide onto the surface of Ti6Al4V alloy could enhance the adhesion and spreading of osteoblasts and further promote the expression of ALP and COL1 genes. The fabrication of FTA could pave the road for designing and building more efficient prothesis for osseointegration within the bone/prosthesis interface in revision THA. And also further* in vivo* experiments are necessary to comprehensively test the biological effect of FTA scaffolds in the future.

## 5. Conclusions 

This study showed that the RGD oligopeptide could be covalently conjugated onto the Ti6Al4V alloy to fabricate the functional Ti6Al4V alloy (FTA). The distribution of RGD oligopeptide on the FTA was even and homogeneous. The FTA scaffolds were more favorable for osteoblast adhesion and spreading. Finally, the FTA scaffolds were more beneficial to osseointegration, which may be due to the improvement of osteoblast adhesion by the RGD oligopeptide coated on the Ti6Al4V alloy. Therefore, the FTA scaffolds developed herein pave the road for designing and building more efficient prothesis for osseointegration between the host bone and prothesis in revision THA.
